# Knowledge, attitudes and practices of cervical cancer prevention among Zambian women and men

**DOI:** 10.1186/s12889-019-6874-2

**Published:** 2019-05-04

**Authors:** Anayawa Nyambe, Jarl K. Kampen, Stridutt K. Baboo, Guido Van Hal

**Affiliations:** 1 Faculty of Medicine and Health Sciences, University of Antwerp, Antwerp, Belgium; 2 Biometris, Wageningen University, Wageningen, Netherlands, and StatUA (Core Facility for Statistical Analysis), University of Antwerp, Antwerp, Belgium; 3 Department of Public Health and Tropical Medicine, University of Zambia, Lusaka, Zambia; 4 Department of Epidemiology and Social Medicine, University of Antwerp, Antwerp, Belgium

**Keywords:** Cervical cancer, Zambia, Screening, Vaccination, Knowledge, Attitude, Practices, Social ecological model, Theory of triadic influence

## Abstract

**Background:**

In Zambia, cervical cancer screening was started in 2006 and the human papillomavirus vaccine was piloted in 2013. Nevertheless, cervical cancer remains the leading cancer. It is assumed that knowledge, social interaction, health behaviors and religion are factors that can influence screening and vaccination practices. This study addresses the question, what is the relationship between knowledge about cervical cancer, attitudes, self-reported behavior, and immediate support system, towards screening and vaccination of cervical cancer of Zambian women and men. The results of this study serve as a basis for future research, an input for improvement and adjustment of the existing prevention program and build on documented health behavior frameworks.

**Methods:**

A cross-sectional mixed methods study was conducted from February to May 2016. Two separate questionnaires were used to collect data from women (*N* = 300) and men (*N* = 300) residing in Chilenje and Kanyama (two townships in the capital city Lusaka). Respondent’s knowledge of cervical cancer was operationalized by grading their ability to correctly identify causes and protective factors if they were aware of cervical cancer. Besides providing descriptive statistics of all study variables, we tested four research hypotheses concerning the link between knowledge, attitudes and practices suggested by the literature, by applying appropriate statistical tests (chi square test, analysis of variance, logistic regression).

**Results:**

Less than half of the respondents (36.8%) had heard of cervical cancer, 20.7% of women had attended screening and 6.7% of the total sample had vaccinated their daughter. Knowledge of causes and prevention was very low. There was a strong association between having awareness of cervical cancer and practicing screening (odds ratio = 20.5, 95% confidence interval = [9.214, 45.516]) and vaccination (odds ratio = 5.1, 95% confidence interval = [2.473, 10.423]). Social interactions were also found to greatly influence screening and vaccination behaviors.

**Conclusions:**

The low level of knowledge of causes and prevention of cervical cancer suggests a need to increase knowledge and awareness among both women and men. Interpersonal interactions have great impact on practicing prevention behaviors, for instance, vaccination of daughters.

**Electronic supplementary material:**

The online version of this article (10.1186/s12889-019-6874-2) contains supplementary material, which is available to authorized users.

## Background

Cervical cancer prevention programs provide services that prevent and reduce mortality of cervical cancer cases. For a health program at the level of a community or a nation to be administered, it must be feasible. Aspects of feasibility include having adequate infrastructure (e.g., equipment, institutions, know-how, etc.) on the one hand, and support from the necessary stakeholders, including women from the target group and their partners, on the other. This study on the topic of cervical cancer and its prevention is part of a larger research project that aims to identify the most optimal prevention and screening procedures practicable in Zambia (see [34]).

In this paper, we focus on Zambian women as stakeholders, assessing the relationship between their knowledge about cervical cancer, attitudes and self-reported behavior towards screening and vaccination, as well as their immediate support system including men, other family members and friends. We noted that existing studies of populations in Zambia are limited in availability and incomplete. The available literature on cervical cancer prevention in Zambia did not cover the acceptability of the vaccination of boys, or the willingness to self-screen, etc. Moreover, while various studies in other countries have assessed factors such as knowledge, social interaction, culture and religion, as well as alternative methods of screening, their applicability for the populations in urban and peri-urban townships in Lusaka (Zambia) is unknown. Accordingly, we conducted an empirical study that covers these and other factors that influence cervical cancer prevention practices in the Zambian populations residing in Lusaka. This extensive data that was gathered and assessed was used to develop an intervention study targeting knowledge and can be used as the basis for the development of other future research.

The development of our instrument (a questionnaire) and the selection of study groups were guided by the Social Ecological Model (SEM) by McLeroy et al. [24], and the Theory of Triadic Influence (TTI) by [13]). The SEM considers intrapersonal, interpersonal, institutional, community and public policy as levels of influence for health related behaviors [24]. The TTI however, is organized in a 3 × 3 framework with intrapersonal, interpersonal and environmental streams of influence, crossed by ultimate, distal and proximal levels of influence [13]. These frameworks though differing in structure and variable interaction, share many theoretical concepts. Thus, allowing them to be integrated in this study. For a systematic review on the SEM and TTI in relation with cervical cancer prevention, see Nyambe, Van Hal and Kampen [34].

### Literature review and hypotheses

Most women are at risk of contracting cervical cancer. Unlike most other cancers, cancer of the cervix is one of the most preventable by both primary and secondary prevention methods. In Zambia, the see-and-treat cervical cancer screening program was started in 2006 initially only targeting Human immunodeficiency virus (HIV) positive women before being made available to all women regardless of HIV status [7, 29]. To enhance coverage, nurses were trained to conduct cervical screening and the electronic cervical cancer control (eC3) was developed to assist their consultation with health professionals using internet and SMS technology [35] Cervical cancer screening via Visual Inspection with Acetic acid (VIA) is provided free of charge at government clinics in every province of the country where screening services are available (Cervical Cancer Prevention Program Zambia, [7, 8]). Then in 2013, the human papillomavirus (HPV) vaccine was launched as a demonstration in Lusaka Province [46], as another means of preventing the spread of cervical cancer in the country. Funded by Gavi, the HPV vaccine Gardasil was administered using a school-based strategy targeting girls in grade 4 (aged 9 to 13 years old) and out of school girls aged 10 years old [46]. Despite these developments, cervical cancer remains the leading cancer in Zambia. According to the Cancer Disease Hospital in Lusaka, approximately 35% of all cancers managed are cervical cancers [25].

Though little research targeting Zambian populations has been published, studies in various other countries have identified factors that can supposedly predict screening and vaccination practices. Research conducted in Uganda [31], Tanzania [11] and South Africa [21], has suggested that one of the reasons women do not practice screening is lack of knowledge of cervical cancer and its prevention. Among Zambian women, familiarity to vaccines in general made acceptance of the HPV vaccine higher for themselves as well as for their daughters [18]. Similarly, having knowledge and awareness of cervical cancer resulted into positive attitudes towards the vaccine among Zambian parents/guardians [26] and Cameroonians [2, 41]. Based on these studies, we assumed that women who know of cervical cancer are more likely to practice screening and agree to vaccination.

Several studies have suggested that support from immediate social circles can influence likelihood of women practicing screening and agreeing to the vaccination of their children. For instance, women in Uganda who know someone who has screened have a higher chance of practicing screening themselves [31]. Research conducted in Tanzania [11] and Nigeria [1, 23], found that decision making was influenced by a woman’s partner/husband, who must support, or help make, the decision to practice prevention. In Zambia, it was found that most women discussed their screening decisions with members of their social network [43]. This evidence suggests that women who believe they have support from their immediate social circles (partner, friends, family) are more likely to be in favor of practicing cervical cancer prevention methods.

Studies in the USA and England have also shown the influence of women’s behavior towards preventive measures on HPV vaccine uptake in their children. It was found that daughters with mothers who practice screening were more likely to be vaccinated than those who had mothers that did not screen [9] or personally decided to stop screening [38]. It was therefore reasonable to assume that women who practice screening are more likely to want to vaccinate their children.

Finally, factors such as religion and cultural beliefs were also identified to influence health practices. A study in Nigeria found that barriers to cervical screening vary by religion [27]. Additionally, a study on school teachers in Kenya found that some religious beliefs were against vaccinations [20]. This made us assume that religious beliefs limit the uptake of screening and vaccination.

## Methods

### Study design, site and population

A cross-sectional study was designed employing both quantitative and qualitative data collection methods. In this study, emphasis is on quantitative data collected by means of a questionnaire. For the results of the qualitative data obtained from stakeholders (health care providers, teachers, religious leaders), special interest groups (advocacy groups, non-governmental organizations, media) and policy makers see Nyambe et al. [32, 33]. The study was carried out from February to May 2016. Inclusion criteria for respondents from the general public to participate in this research were that they must: i) be a resident of Lusaka City in Chilenje or Kanyama, ii) be at least 18 years old, and iii) have at least one primary/secondary school going child.

The target population of women and men from the general public resided in either Chilenje Township or Kanyama Compound of Lusaka City. These two neighborhoods were selected because of their comparable availability of cervical screening services and population density, and their difference in living standards (Chilenje being relatively richer than Kanyama [19]). These respondents who included women and men of Chilenje or Kanyama were also a parent/legal guardian of at least one child in primary or secondary school. This was because the vaccine was administered in a school-based program. Children in higher education (college, university) are in most cases considered adults, and are able to decide on having a vaccination without parental consent. The identities of the respondents and the information they provided were treated as confidential. Approval for conducting this study was obtained from Eres Converge (Lusaka, Zambia).

Conventional strategies for computing sample size in empirical studies lead to the conclusion that a sample of size *n* = 100 (50 in each group) would be sufficient to detect a difference between two groups of 1 standard deviation with 99% power at 1% level of significance by an independent sample t test (see e.g., [10]). For the purpose of our survey, it was decided to take a much bigger sample that would cover the heterogeneity of the target population in terms of relative wealth, gender, education and levels of knowledge. The sample size was disproportionally stratified for the number of households in Chilenje and Kanyama. The Zambian Demographic Health Survey of 2007 defined a household as “a person or a group of persons, related or unrelated, who live together and share common cooking and eating arrangements.” In Zambia, about three quarters of households are male headed, and the remaining are female headed [5]. The total numbers of households in Chilenje was 10,330 according to the last official national census of 2010, and 35,682 households in Kanyama [4]. After considering the variables under study and likely response rate, we decided on a sample size of 100 women and 100 men in Chilenje, and 200 women and 200 men in Kanyama.

### Questionnaires and measures

Two separate but similar questionnaires administered by face-to-face interview, were developed for female and male respondents. The questionnaires were designed based on the indicators of health behavior as suggested by the TTI and SEM at the intrapersonal, interpersonal and environmental levels. To combine these study frameworks, the environmental level integrated the institutional, community and public policy levels of the SEM. Each question was either adapted from other studies or developed by the authors of this research.

To ease application, the wide range of information covered in the questionnaires, was arranged into the sections demographics (14 items at intrapersonal, interpersonal and environmental levels), general information (8 items at intrapersonal and interpersonal levels), cervical cancer (35 items for females, 34 items for males, with indicators at intrapersonal, interpersonal and environmental levels), and interview quality assessment (5 items). The section on cervical cancer was subdivided into (a) general cervical cancer, (b) cervical cancer screening, and (c) cervical cancer vaccination. At the end of the sub-section general cervical cancer, all respondents were given basic information on cervical cancer to bridge the knowledge gap. In regard to cervical cancer screening, female respondents were probed on their screening practices while male respondents were directed toward their support role and ability to enable their partner to practice screening. The cervical cancer vaccination section focused on the views of the parents towards vaccinating their children and not themselves.

Interview quality assessment questions were answered by the interviewer to help determine the quality of the interview. These questions were either adapted from the World Value Survey by Nevitte [30] or developed by the authors. They included assessing the interest of the respondent [30], privacy of the interview [30], if it ran smoothly, if problems were faced, and if they had any other remarks. Additional files 1 and 2 have the complete female and male questionnaires respectively [see Additional file 1 and Additional file 2].

The indicators of behavior at intrapersonal level under consideration included age, education level [30], awareness of cervical cancer, knowing causes and protective factors, sources of information, screening attendance, having a vaccinated daughter, willingness to vaccinate children. Interpersonal level items included, living status [16], knowing someone who practices screening [17], accompanying someone for screening [17], perceived approval and disapproval of members of the social circle towards screening and vaccination, knowing someone who has gone for vaccination [17], and approval of others regarding vaccination. Lastly, environmental level items included employment, money use [30], religion and frequency of attending religious meetings [30], importance of religion in daily life [3], knowledge about the availability of screening services, knowing that the government provided free screening services, and knowing the government provided HPV vaccination to school girls.

### Operationalization of knowledge of cervical cancer

Knowledge of cervical cancer was only assessed if a respondent said that they had awareness of cervical cancer which is merely stating that one knows about or has heard about cervical cancer. The knowledge of the respondent with respect to the possible causes (HPV infection, practicing unsafe sex, becoming sexually active at a young age, having a Sexually Transmitted Infection (STI), having many sexual partners, smoking, using contraceptives, vaginal douching) and protective factors (attending regular screening, HPV vaccination, practicing safe sex, being circumcised, not becoming sexually active at a young age, being faithful to one sexual partner, not smoking, not taking contraceptives, no vaginal douching) of cervical cancer was given a knowledge grade ranging from 1 to 10 points. These causes and protective factors of cervical cancer were identified on the basis of the scientific literature ([44] & [45]; [6, 22, 12]). For further information on the calculation of the knowledge grade, see Nyambe et al. [32, 33].

### Sampling design

To avoid problems with illiteracy and possible language barriers, the questionnaires were administered as an interview by data collectors. Four data collectors were assigned to Kanyama and two data collectors to Chilenje. The data collectors worked male and female in pairs, with the male data collector collecting data from men and the female from women. Men and women were interviewed separately. Effort was made to interview both husband and wife, but this failed because of the general lack of interest in men to participate and the unavailability of husbands at home due to work or other reasons for absenteeism. Single parents were not excluded from the research. All respondents were read an information sheet about the study and signed a consent form. Participation was voluntary.

Due to inadequate naming of roads and house numbers, sampling was conducted using the random walk technique [40]. Maps of Chilenje and Kanyama were provided to the data collectors. The main roads were divided into sections, the data collectors would start from a section of the road and work their way inside the community using a spinning pointer when at crossroads. Data collection was targeted at every second house on a road. The data collectors were also equipped with GPS devices from the University of Zambia, School of Mines, which they used to mark every house they collected data from on the map. This ensured that they covered a great area of the townships. Data collectors knocked on doors or gates to ask parents if they had time and were interested in participating in the research. A leaflet with information on cervical cancer from the local clinic was given to all study respondents after the interview as a form of educating the public. The effect of handing out these leaflets was not evaluated in this study.

### Data analysis

To summarize and characterize the data, descriptive statistics of categorical data were reported in frequencies and percentages. Chi-square tests and Fisher’s exact tests were used to report between-group comparisons of categorical variables. Odds ratios (OR) were given for chi-square tests with binominal data. Ordinal variables were expressed using the median. Means and Standard Deviation (SD) with 95% confidence intervals (CI) were used to report normally distributed continuous variables. Between-group comparisons of continuous variables were done using independent samples t-tests. Binominal logistic regression analyses were conducted to find the most important predictors identified in the bivariate analyses of the dependent variables “having practiced screening” i.e. since inception of screening services, “having a vaccinated daughter” i.e. during the running of the pilot vaccination program, “intention to vaccinate a daughter”, and “intention to vaccinate son”. The independent variables were included in the regression equation by forward Wald. To see if accessibility of services affects practice, distances between houses and local clinic were calculated using Google Maps [14]. The locations were mapped using Global Positioning System (GPS) devices in the Universal Transverse Mercator (UTM) coordinate format which was converted to latitude and longitude coordinates using GPS Geo Planner [15]. To control for capitalization on chance, the statistical significance level was set at a relatively conservative level of 1%. All quantitative data was entered in excel and then transferred to Statistical Package for the Social Sciences, IBM SPSS 23 software for windows where it was checked for data that was incorrectly entered or skipped.

## Results

### Sample composition and practices

The sociodemographic characteristics and cervical cancer practices of the respondents are summarized in Table 1 . The respondents were recruited from Chilenje and Kanyama, and their ages were distributed between 18 and 67 years (*M* = 35.9, *SD* = 9.09). The population aged 15 to 64 years is 59.1% of the urban population of Lusaka Province [4]. In the sample, as to level of education, the largest group (26.8%) had attended secondary school without completion, 23.0% had completed higher secondary education, and 7.2% had obtained a university degree. Two out of five (43.3%) respondents were unemployed; students (*N* = 5, 0.8%), retired/pensioned (*N* = 9, 1.5%) were included into category ‘other’ (3.3%). Over half of the respondents (61.8%) were Christian Protestants, and 20.3% were Catholics. Other religious groups (2.2%) included orthodox (*N* = 1, 0.2%) and Muslim (*N* = 7, 1.2%). The majority (63.2%) were not aware of cervical cancer. Many women had not attended cervical screening (79.3%) and furthermore, 93.3% of total population had reported not having their daughters vaccinated. When it came to having interest in vaccinating children in the future if given a chance, 82.7% said they would vaccinate their daughters and 84.0% would vaccinate their sons. Both women and men showed an interest in having the possibility of self-screening with at total of 80.2%. The results for interest in self-screening and distance to the clinic, showed no significance.

**Table 1:** Socio-demographic information

		Total sample (*N* = 600)	Kanyama (*N* = 400)		Chilenje (*N* = 200)	
		%	Female%	Male%	Female%	Male%
Age	24 years and below	9.7	20.5	7.0	3.0	0.0
	25–29 years	16.3	21.0	17.5	21.0	0.0
	30–34 years	21.8	18.0	28.0	17.0	22.0
	35–39 years	19.8	12.0	19.0	23.0	34.0
	40–44 years	17.2	15.0	16.0	19.0	22.0
	45–49 years	8.7	6.0	7.0	12.0	14.0
	50–54 years	2.7	3.0	1.5	1.0	6.0
	55 years and above	3.8	4.5	4.0	4.0	2.0
	Total N (%)	600 (100.0)	200 (100.0)	200 (100.0)	100 (100.0)	100 (100.0)
Education level	No formal education	3.7	3.5	4.0	3.0	4.0
	Incomplete primary school	9.8	19.5	9.0	1.0	1.0
	Complete primary school	12.7	18.0	18.0	2.0	2.0
	Incomplete secondary school	26.8	46.5	25.5	6.0	11.0
	Complete secondary school	23.0	10.5	36.5	23.0	21.0
	Incomplete college, without diploma	3.7	0.0	1.0	11.0	9.0
	Complete college, with diploma	10.2	1.5	5.5	22.0	25.0
	Some university-level education, without degree	3.0	0.0	0.5	10.0	7.0
	University-level education, with degree	7.2	0.5	0.0	22.0	20.0
	Total N (%)	600 (100.0)	200 (100.0)	200 (100.0)	100 (100.0)	100 (100.0)
Employment	Full time employee	20.5	6.0	18.5	33.0	41.0
	Part time employee	9.5	0.0	18.0	9.0	12.0
	Self employed	23.3	19.0	38.0	10.0	16.0
	Unemployed/ housewife	43.3	71.0	23.5	44.0	27.0
	Other	3.3	4.0	2.0	4.0	4.0
	Total N (%)	600 (100.0)	200 (100.0)	200 (100.0)	100 (100.0)	100 (100.0)
Income	Save money	28.3	17.5	46.0	17.0	26.0
	Just get by	74.0	83.5	58.0	80.0	81.0
	Spent some savings	7.8	7.5	2.0	11.0	17.0
	Spent savings and borrowed money	3.3	1.0	4.0	4.0	6.0
	Total N (%)	600 (100.0)	200 (100.0)	200 (100.0)	100 (100.0)	100 (100.0)
Religion	None	15.7	1.5	16.5	24.0	34.0
	Catholic	20.3	9.0	17.0	36.0	34.0
	Christian Protestant	61.8	89.0	62.0	37.0	32.0
	Other	2.2	0.5	4.5	3.0	0.0
	Total N (%)	600 (100.0)	200 (100.0)	200 (100.0)	100 (100.0)	100 (100.0)
Living Status	Alone	3.3	1.0	5.5	0.0	7.0
	Partner	71.2	61.5	78.0	65.0	83.0
	Stepchildren	4.3	3.0	3.5	6.0	7.0
	Bio-children	83.3	82.0	78.0	88.0	92.0
	Other children	43.0	43.0	16.0	69.0	71.0
	Other family	46.7	29.5	45.0	71.0	60.0
	Other	0.7	1.0	0.5	1.0	0.0
	Total N (%)	599 (99.8)	200 (100.0)	199 (99.5)	100 (100.0)	100 (100.0)
Do you know cervical cancer? (awareness)	Yes	35.8	33.5	36.5	46.0	29.0
	No	63.2	66.0	63.5	51.0	69.0
	Not sure	1.0	0.5	0.0	3.0	2.0
	Total N (%)	600 (100.0)	200 (100.0)	200 (100.0)	100 (100.0)	100 (100.0)
Have you screened?	Yes	20.7	17.0	–	28.0	–
	No	79.3	83.0	–	72.0	–
	Total N (%)	300 (100.0)	200 (100.0)	–	100 (100.0)	–
Interest in self-screening	Yes	80.2	83.5	84.5	79.0	69.0
	No	13.0	14.0	9.5	19.0	12.0
	Not sure	6.8	2.5	6.0	5.0	19.0
	Total	600 (100.0)	200 (100.0)	200 (100.0)	100 (100.0)	100 (100.0)
Have you vaccinated your daughter?	Yes	6.5	5.5	4.0	10.0	10.0
	No	93.3	94.5	95.5	90.0	90.0
	Total N (%)	599 (99.8)	200 (100.0)	199 (99.5)	100 (100.0)	100 (100.0)
Future vaccination of daughter	No	4.7	4.5	9.0	1.0	0.0
	Yes, if given a chance	82.7	83.0	79.5	86.0	85.0
	I do not have a daughter but if I did I would vaccinate her	6.7	7.0	7.5	3.0	8.0
	Total N (%)	564 (94.0)	189 (94.5)	192 (96)	90 (90.0)	93 (93.0)
Vaccination of son	No	7.0	6.0	10.5	5.0	4.0
	Yes, if given a chance	84.0	84.0	78.0	90.0	90.0
	I don’t have a son but if I did I would vaccinate him	8.0	9.0	10.0	5.0	5.0
	I don’t have a son but if I did I would not vaccinate him	0.3	0.0	0.5	0.0	1.0
	Total N (%)	596 (99.3)	198 (99.0)	198 (99.0)	100 (100.0)	100 (100.0)

Factors that could potentially affect the quality of the interviews varied depending on the area the respondents resided. In Kanyama, 81.5% of the respondents were very interested in the interview compared to Chilenje were only 23.5% were very interested. A substantial part of the respondents in Chilenje were somewhat interested (*N* = 80, 40.0%). The data collectors in both areas made an effort to ensure that interviews were conducted in private with no other people (*N* = 397, 66.2%) and they equally reported the interviews to have run smoothly (*N* = 559, 93.2%). Data collectors stated that the difficulties they faced were due to others intruding while the interview was taking place, language barriers, respondents expecting further assistance because they participated, and respondents general non-interest or ignorance of the topic which greatly lengthened the time of the interview.

### Knowledge, practices and intentions

Table 2 reports the relationship between awareness of cervical cancer and practicing screening and having vaccinated the daughter. Having awareness of cervical cancer and conducting these practices, shows strong significance. Screening accompaniment refers to whether a woman had an escort or was invited to be taken for screening. There was an association between being aware of cervical cancer and being invited or inviting someone for screening . It was further found that the greater majority of the population that is aware of cervical cancer and its prevention services will vaccinate their children if given a chance in the future. Of the very few parents who expressed disinterest in vaccinating children in the future, the majority of them resided in Kanyama. In Chilenje, all men expressed interest in vaccinating their daughters. Significant associations were found for women who know about screening at government clinics and future vaccination of daughter (χ^2^ = 12.8, *df* = 2, *p* < 0.01), women who know about schools that vaccinated and future vaccination of daughter (χ^2^ = 15.6, *df* = 4, *p* < 0.01), men who know about screening in government clinics and vaccination of sons (χ^2^ = 12.3, *df* = 3, *p* < 0.01).

**Table 2:** Awareness of cervical cancer and knowing about prevention services in relation to practicing prevention

	Yes	No	Chi-square test				
			OR	95% CI	χ^2^	*df*	*p*
Have you screened^*^							
Do you know cervical cancer (awareness)							
Yes	54	59	20.5	[9.214, 45.516]	81.3	1	0.000
No	8	179					
Know where to screen							
Yes	58	89	24.3	[8.523, 69.140]	62.1	1	0.000
No	4	149					
Know about screening in clinics							
Yes	58	91	23.3	[8.170, 66.246]	59.8	1	0.000
No	4	146					
Know about vaccination of school girls							
Yes	28	28	6.2	[3.267, 11.678]	36.1	1	0.000
No	34	210					
Know schools that participated in vaccination							
Yes	17	18	4.6	[2.211, 9.642]	18.8	1	0.000
No	45	220					
Screening accompaniment							
Do you know cervical cancer (awareness)							
Female							
Yes	17	45	8.5	[1.855, 38.954]	10.0	1	0.002
No	2	45					
Male							
Yes	17	85	13.0	[3.711, 45.535]	24.8	1	0.000
No	3	195					
Know where to go for screening							
Female							
Yes	17	51	6.5	[1.417, 29.818]	7.2	1	0.007
No	2	39					
Male							
Yes	15	76	8.1	[2.830, 22.916]	20.2	1	0.000
No	5	204					
Know about screening in clinics							
Female							
Yes	18	50	14.0	[1.795, 109.799]	10.0	1	0.002
No	1	39					
Male							
Yes	16	100	7.2	[2.343, 22.124]	15.4	1	0.000
No	4	180					
Know about vaccination of school girls							
Female							
Yes	10	22	3.4	[1.237, 9.532]	6.0	1	0.014
No	9	68					
Male							
Yes	6	65	1.4	[0.524, 3.837]	0.5	1	0.490
No	14	215					
Know schools that participated in vaccination							
Female							
Yes	7	13	3.5	[1.148, 10.400]	5.3	1	0.022
No	12	77					
Male							
Yes	6	24	4.6	[1.610, 12.984]	9.5	1	0.002
No	14	256					
Have vaccinated their daughter							
Do you know cervical cancer (awareness)							
Female							
Yes	17	96	8.1	[2.652, 24.752]	18.0	1	0.000
No	4	183					
Male							
Yes	11	91	3.3	[1.231, 8.742]	6.2	1	0.013
No	7	190					
Know where to go for screening							
Female							
Yes	18	129	7.0	[2.009, 24.223]	12.2	1	0.000
No	3	150					
Male							
Yes	8	82	1.9	[0.740, 5.094]	1.9	1	0.171
No	10	199					
Know about screening in clinics							
Female							
Yes	20	129	23.1	[3.058, 174.516]	18.6	1	0.000
No	1	149					
Male							
Yes	10	106	2.1	[0.790, 5.392]	2.3	1	0.132
No	8	175					
Know about vaccination of school girls							
Female							
Yes	16	40	19.1	[6.634, 55.107]	49.2	1	0.000
No	5	239					
Male							
Yes	9	62	3.5	[1.344, 9.281]	7.3	1	0.007
No	9	219					
Know schools that participated in vaccination							
Female							
Yes	12	23	14.8	[5.661, 38.908]	46.3	1	0.000
No	9	256					
Male							
Yes	5	25	3.9	[1.298, 11.953]	6.7	1	0.010
No	13	256					

*Only women asked. (No/not sure) for all variables

Strong associations were found regarding respondent’s awareness of cervical cancer in relation to presumed support and possible support provided by their partner to allow screening attendance. For partner’s support, the categories ‘completely disagree’, ‘disagree’ and ‘neither’ were combined due to scarcity of results. Results were significant for awareness of cervical cancer and partners support when responses ‘no’ and ‘not sure’ were combined due to scarcity (female respondents median = 2, χ^2^ = 24.4, *df* = 1, *p* < 0.01; male respondents median = 2, χ^2^ = 9.4, *df* = 1, *p* < 0.01), for women who know where to go for screening and partners support when responses ‘no’ and ‘not sure’ were combined due to scarcity (median = 2, χ^2^ = 64.3, *df* = 1, *p* < 0.01), and for women who know about screening in government clinics (median = 2, χ^2^ = 63.8, *df* = 1, *p* < 0.01).

The results of the knowledge grade analysis are summarized in Table 3. The knowledge grade of men (*M* = 4.24) was slightly higher than of women (*M* = 3.03), but grades were very low relative to maximum of 10 that could be achieved if all causes and protective factors are correctly identified. The most commonly correctly identified causes are “having many sexual partners” (correctly identified by 42% of women and 60% of men), “practicing unsafe sex” (correctly identified by 38% of women and 50% of men, “HPV infection” (correctly identified by 38% of women and 44% of men), and “vaginal douching” (correctly identified by 47% of women and 41% of men). The most commonly correctly identified protective factors are “no vaginal douching” (correctly identified by 40% of women and 49% of men), “being circumcised” (correctly identified by 43% of women and 56% of men), and “attending regular screening” (correctly identified by 51% of women and 47% of men). The other causes and protective factors are only sporadically correctly identified. Quite surprisingly, the degree of knowledge (by grade or separate factors) does not differ between those screened and did not screen, or those that vaccinated or did not vaccinate.

**Table 3:** Means (sd) of cervical cancer Knowledge Grades by gender, screening and vaccination*

	Gender		Screening practice		Vaccination practice	
	Female (*N* = 117)	Male (*N* = 104)	Yes (*N* = 54)	No (*N* = 63)	Yes (*N* = 28)	No (*N* = 193)
Causes						
Smoking	.06 (.24)	.14 (.35)	.07 (.26)	.05 (.21)	.04 (.19)	.11 (.31)
Becoming sexually active at a young age	**.21 (.41)**	**.52 (.50)**	.20 (.41)	.22 (.42)	.29 (.46)	.37 (.48)
Having many sexual partners	**.42 (.50)**	**.60 (.49)**	.46 (.50)	.38 (.49)	.46 (.51)	.51 (.50)
Becoming pregnant at a young age	**.02 (.13)**	**.14 (.35)**	.00(.00)	.03 (.18)	.04 (.19)	.08 (.28)
Using contraceptives	.05 (.22)	.10 (.30)	.00 (.00)	.10 (.30)	.04 (.19)	.08 (.27)
Having many pregnancies	.03 (.19)	.12 (.32)	.00 (.00)	.06 (.25)	.04 (.19)	.08 (.27)
Being old	.03 (.18)	.02 (.14)	.04 (.19)	.03 (.18)	.04 (.19)	.03 (.16)
Vaginal douching	.47 (.50)	.41 (.49)	.52 (.50)	.43 (.50)	.61 (.50)	.42 (.49)
Heredity	.03 (.18)	.06 (.23)	.02 (.14)	.05 (.21)	.07 (.26)	.04 (.20)
Having a STI	**.23 (.42)**	**.44 (.50)**	.28 (.45)	.19 (.40)	.43 (.50)	.32 (.47)
Practicing unsafe sex	.38 (.49)	.50 (.50)	.44 (.50)	.33 (.48)	.54 (.51)	.42 (.50)
HPV infection	.38 (.49)	.44 (.50)	.50 (.50)	.29 (.46)	.54 (.51)	.39 (.49)
Protective Factors						
Not smoking	**.05 (.22)**	**.17 (.38)**	.06 (.23)	.05 (.21)	.04 (.19)	.12 (.32)
Not becoming sexually active at a young age	**.18 (.39)**	**.55 (.50)**	.17 (.38)	.19 (.40)	.25 (.44)	.37 (.48)
Being faithful to one sexual partner	**.29 (.46)**	**.58 (.50)**	.35 (.48)	.24 (.43)	.54 (.51)	.41 (.49)
Not taking contraceptives	**.01 (.09)**	**.10 (.30)**	.00 (.00)	.02 (.13)	**.00 (.00)**	**.06 (.23)**
No vaginal douching	.40 (.48)	.49 (.50)	.50 (.50)	.32 (.47)	.57 (.50)	.42 (.49)
Practicing safe sex	**.24 (.43)**	**.51 (.50)**	.28 (.45)	.21 (.41)	.39 (.50)	.36 (.48)
Being circumcised	.43 (.50)	.56 (.50)	.44 (.50)	.41 (.50)	.57 (.50)	.48 (.50)
HPV vaccination	.36 (.48)	.45 (.50)	.48 (.50)	.25 (.44)	.54 (.51)	.38 (.49)
Attending regular screening	.51 (.50)	.47 (.50)	.59 (.50)	.44 (.50)	.46 (.51)	.50 (.50)
Knowledge grade	**3.03 (2.69)**	**4.24 (2.81)**	3.55 (2.64)	2.58 (2.67)	4.02 (2.80)	3.54 (2.81)

*Numbers in bold signify statistical significance of t-test at *p* < 0.01. Only women were asked about practicing screening. Respondent who said they did not hear about cervical cancer were not asked about causes and protective factors

Respondents in Kanyama, reported that their information on cervical cancer was mostly obtained from healthcare providers (*N* = 93, 66.0%) and television/radio (*N* = 73, 51.8%). Equally, in Chilenje healthcare providers provided most information (*N* = 62, 78.5%), followed by internet (*N* = 28, 35.4%). The majority of the respondents identified healthcare providers (*N* = 576, 96.2%), internet (*N* = 256, 42.7%) and television/radio (*N* = 249, 41.6%) as good sources to gain future information on cervical cancer.

### Impact of social support on practice and intentions

Women who practiced screening were highly likely to also know someone who has screened (OR = 18.7, 95% CI = [9.270, 37.554], χ^2^ = 91.3, *df* = 1, *p* < 0.01). Regarding perceived approval of practicing a preventive measure, we found strong associations between perceived approval of screening and actually practicing screening: women are much more likely to have practiced screening if they enjoy support of their partner (χ^2^ = 11.4, *df* = 1, *p* < 0.01), family (χ^2^ = 11.8, *df* = 1, *p* < 0.01), or friends (χ^2^ = 8.6, *df* = 1, *p* < 0.01). The intention to vaccinate daughters in the future if given a chance, is positively related with women’s perceived approval from their partners (χ^2^ = 10.8, *df* = 2, *p* < 0.01). Intention to vaccinate sons was not significantly associated with support of any kind. For both men (OR = 14.2, 95% CI = [4.889, 41.166], χ^2^ = 36.4, *df* = 1, *p* < 0.01) and women (OR = 29.0, 95% CI = [10.402, 80.852], χ^2^ = 74.3, *df* = 1, *p* < 0.01), the probability of having daughters vaccinated increases when knowing someone who has vaccinated. No relationship between practicing vaccination of daughters and social support was found. When it comes to intentions to vaccinate, there is a significant relationship between women who know someone who has practiced vaccination and the intention of vaccinating their daughter in the future if given a chance (χ^2^ = 15.8, *df* = 2, *p* < 0.01). No significant relationship was found between women who know someone who has vaccinated and the intention to vaccinate sons in the future.

Concerning the relationship between women who practice screening and whether they are willing to vaccinate their children, there was a strong relationship found between women who have screened and have vaccinated their daughters (OR = 9.6, 95% CI = [3.689, 25.114], χ^2^ = 29.1, *df* = 1, *p* < 0.01). In fact, 22.6% of women had vaccinated her daughter if she herself was screened, against 2.9% if she herself had not screened. On the other hand, whether women practiced screening or not showed no association with interest in vaccinating daughters in the future and the vaccination of sons.

### Impact of religious beliefs on knowledge, practice and intentions

Religion in relation to practice of screening suggested no significance. A respondent’s religious affiliation was significant in terms of having a vaccinated daughter (χ^2^ = 16.2, *df* = 3, *p* < 0.01). Furthermore, unlike religious affiliation, the more important a respondent regarded religion determined their intention to vaccinate their son in future, (χ^2^ = 33.8, *df* = 12, *p* < 0.01). Religious importance and having heard of cervical cancer also showed significance, (χ^2^ = 24.7, *df* = 8, *p* < 0.01). A total of 33 respondents stated that they had gained information on cervical cancer from their religious group. Where 30 of them recalled the different types of information they acquired, which included information on cervical screening (*N* = 15, 50.0%), vaccination (*N* = 7, 23.3%), abstinence from sex (*N* = 7, 23.3%), and practice of safe sex (*N* = 3, 10.0%).

### Multivariate analysis

A logistic regression analysis use general social demographic characteristics (gender, age, education, employment, income, religion, living status), awareness (knowing/having heard about cervical cancer, knowing where women can go for screening, knowing government clinics provide free screening, knowing about the HPV vaccine, knowing about vaccination in schools), knowing someone who has screened or vaccinated, being accompanied for screening, perceived approval of screening (from partner, family and friends), and vaccination approval from partners. The knowledge grade was not included in the regression analysis because it was not calculated for respondents who were not aware of cervical cancer and since these were the majority it would cause error in the overall analysis. Predictors of behavior were found for screening, having a vaccinated daughter and future vaccination of daughters. There were no significant results for the outcome of intention to vaccinate sons in the future if given a chance.

Significant predictors for the outcome of practicing screening, were “having heard about cervical cancer” (Wald 16.1, *df* = 2, *p* < 0.01), the interpersonal indicators of “knowing someone who has practiced screening” and “living with other children (not biological or step-children)”. For the outcome of having had a vaccinated daughter, the interpersonal indicators of “living with other children” and “knowing someone who has vaccinated” are statistically significant. When it came to intention to vaccinate daughters, perceived vaccination approval from partners (Wald 28.7, *df* = 4, *p* < 0.01) contributed to the equation overall. Such that, when a respondent perceived that their partner would agree to have their daughter vaccinated, there was a higher chance of willingness to have their daughter vaccinated in the future if given a chance. Living with others who are not family (e.g. friends), reduced the chance of intending to vaccinate daughters in the future. Table 4 below, summarizes the significant results of the regression analysis.

**Table 4:** Summary of significant logistic regression results

Predictor	Screening^a^			Vaccination of daughter(s)			Future vaccination of daughter(s)		
	β	OR	95% CI	β	OR	95% CI	β	OR	95% CI
Know someone who has screened	2.571	13.1	[4.042, 42.308]	–	–		–	–	
Living with other children	1.451	4.3	[1.531, 11.883]	1.432	4.2	[1.753, 10.007]	–	–	
Know someone who has vaccinated	–	–		3.257	26.0	[10.780, 62.624]	–	–	
Living with others	–	–		–	–		−4.2	0.0	[0.001, 0.310]
Support from partner to vaccinate (completely disagree)	–	–		–	–		−5.2	0.0	[0.000, 0.158]
Pseudo R2	0.612			0.310			0.247		
% cor pred.	87.7			94.2			95.3		

^a^Only women were asked about practicing screening

## Discussion

### Knowledge, practices and intentions

The presumption was that, women who know of cervical cancer are more likely to practice screening and agree to vaccination. The results in Table 2 support the hypothesis when a respondent says they are aware of cervical cancer and know places providing preventive services. This was the case for both women and men. In particular, men who stated they are aware of cervical cancer were more likely to provide support to their partners in practicing screening. The overall awareness of respondents was low, which is similar to previous studies in Zambia [28, 37]. These results also support the findings of a systematic review that stated there are high levels of willingness to vaccinate but low levels of awareness and knowledge of cervical cancer in sub-Saharan Africa [36].

In our study, it was only the few respondents who stated they were aware of cervical cancer that were asked further questions on knowledge of causes and preventive factors. As seen from Table 3, men were found to have a slightly higher level of knowledge of cervical cancer than women. Men were difficult to recruit into the study and perhaps it was men who were aware of cervical cancer that were more likely to agree to participate. Having knowledge of cervical cancer causes and protective factors as calculated by the knowledge grade, did not show any association with the practice of preventive measures. In depth knowledge of cervical cancer may not be necessary since having awareness is enough for a respondent to practice prevention. Reverse casualty may be assumed in that having practiced prevention, knowledge levels are increased.

There is no denying that general knowledge is fundamental in increasing the practice of prevention methods. This is in accordance to the regression results for the SEM at intrapersonal level where having heard about cervical cancer was a main predictor of behavior. Community-based sensitization strategies have proven to be successful in raising awareness, knowledge and prevention practices [42]. Healthcare providers, internet and television/radio were identified as good sources of information and may provide possible targets for conducting interventions aimed at increasing cervical cancer knowledge in Zambia.

### Impact of social support on practice and intentions

It was assumed that, women who believe they have support from their immediate social circles (partner) are more likely to be in favor of practicing cervical cancer prevention methods. The results support this hypothesis regarding women practicing screening. For vaccination practices, this hypothesis is supported only in the decision making of women concerning vaccinating daughters in the future. It was found that women’s perceived approval of partners, family and friends influenced their screening practices. This finding is similar as in a study in Zambia which determined that women’s decision to screen was often prompted by peers and family members [43]. Other studies [1, 11, 23], also suggested the influence of husbands on decision making. Furthermore, unlike men, only women were more likely to want to vaccinate their daughters if they thought their partners would approve. It can be suggested that there is a relationship between practicing preventive measures and having support especially for women. This implies that Zambian society may be a rather patriarchal society, where men have a big impact in the household. Meaning that for effectiveness of cervical cancer prevention programs, men must be included as a target population.

In addition, there was an association between knowing someone who has practiced screening and actually screening as well as knowing someone who has practiced vaccination and actually vaccinating daughters. This corresponds with the results of the study by Ndejjo, et al. [31], which found that knowing someone who had ever been screened was a predictor of cervical screening. This suggests that interaction with people who practice certain behaviors can influence behavior such that anyone associated with the group behaves according to the group. This hypothesis is further supported by the results of the regression analysis in Table 4, which indicates that living conditions, social support and influence are the overall main predictors of practice of prevention behaviors as suggested by the SEM and TTI at interpersonal level.

Finally, data provided for the evidence of validity of the hypothesis that women who practice screening are more likely to want to vaccinate their children. It was found that there was a strong relationship between women who practice screening and had their daughters vaccinated. Apparently, these women, who are obviously aware of cervical cancer, also see the importance of vaccinating their children.

### Impact of religious beliefs on knowledge, practice and intentions

It was assumed that, religious beliefs limit the uptake of screening and vaccination. The findings go against this theory in that religion showed no influence on screening decisions and showed a positive influence on vaccination acceptance. This is contrary to other countries where religion was found to limit the uptake of vaccines [20, 39].

A total of 82.1% of the population professed to be Christians and this increased the chances of having a vaccinated daughter. The more important the respondent regarded their religion proved to be a possible indicator of intending to vaccinate sons. This suggests that churches in Zambia may play a role in improving vaccination practices. One possible explanation is that some Christian denominations are known to actively educate their members on health issues including cervical cancer. These interpretations are limited because the Christian denominations of the respondents were not assessed. Beliefs between different churches vary. Equally, the accuracy of the information provided by the churches needs to be further investigated.

## Conclusions

The relationship of knowledge, attitudes and self-reported behavior of Zambians residing in Chilenje and Kanyama towards screening and vaccination of cervical cancer is complicated. While factual knowledge of cervical cancer among them is low, knowing about cervical cancer does increase both probability of the women practicing screening, and the intention to have daughters vaccinated. When women had actually screened for cervical cancer the odds that they vaccinated their daughters increased tenfold. When we consider that the most important actor in the immediate social circle of a young girl is her mother, this strongly supports the hypothesis that social interaction is an important factor for the practice of cervical cancer prevention.

Many Zambians in our sample attest to belong to the Christian faith. In most cases churches are accused of rejecting modern medicine due to moral and biblical beliefs. With support from the clergy vaccine coverage can be improved, as is evident from having a higher number of vaccinated daughters belonging to Christian families.

Central clinics with screening services were accessible for women in Chilenje and Kanyama but there was still low attendance. Awareness and ultimately knowledge was found to be one of the main factors for non-attendance considering the availably of accessible and free screening services. Positive attitudes towards self-screening suggest another possible way to maximize coverage for cervical cancer screening. Granted, outreach programs would be necessary to educate the public on cervical cancer and self-sampled screening. Vaccination was only available to grade 4 girls and therefore, not only knowledge, but availability may have affected the low coverage. Nonetheless, the high level of interest in vaccination shows that a full-scale country wide vaccination program may be successful if launched in the future. Identifying this lack of awareness and knowledge is an important step for implementing a successful primary and secondary cervical cancer prevention program focused on increased screening practice and vaccine administration.

Low awareness and knowledge of cervical cancer and cervical cancer prevention highlight the need for sensitization of the general public in order to emphasize the importance of vaccination and screening for cervical cancer. Men were found to influence the practices of women and the acceptability of the HPV vaccine. Interventions involving public health communication on cervical cancer should be targeted to both women and men. Outreach should also be made to churches to ensure the correct information on prevention is disseminated and to encourage church-goers to practice cervical cancer prevention. Considering the low level of awareness and consequently knowledge obtained in this study, an intervention study targeting women and men in churches aimed at enhancing knowledge levels among Zambians living in Lusaka was conducted as part of our larger research project.

The frameworks provided by the SEM and TTI were useful in providing a basis for instrument development and selection of study groups. The main predictor of behavior found at intrapersonal level was having awareness of cervical cancer. Based on the SEM, interventions aimed at improving screening should aim at increasing awareness of cervical cancer. Knowledge influences behavior but most predictors were at the interpersonal level for both the SEM and TTI. Knowing people who have practiced cervical cancer prevention methods and support from family play a role in influencing the health behaviors of a society. According to the SEM and TTI, the impact of the people a person may live with should be considered because they may influence whether screening and vaccination will be practiced. Once people are aware of cervical cancer, perhaps by observing the actions of others, practice of prevention methods will increase resulting in a reduction in the spread of this cancer.

### Limitations and outlook

There are limitations to the findings of this study. Participation in the study was voluntary. Therefore, the majority of the respondents may have been those who showed more interest in the topic. Since the questionnaires were not self-administered, it may have led to dishonesty in respondents because cervical cancer may be considered a sensitive topic. The study participants were limited to parents/guardians of at least one school going child. This helped reduce asking respondents without children questions that are hypothetical but not all respondents had both daughter and son which may have affected the results. Therefore, questions referring to intention to vaccinate were included to cover parents who did not have a chance to vaccinate their children. These questions on intention to vaccinate have not been followed-up to see whether respondents will actually practice what they said. In addition, it was also not assessed if women had intentions to practice screening or continue practicing screening in the future. A follow up study could be considered to assess respondent’s actual future behavior.

Another limitation would be restricting the location of the study to Chilenje and Kanyama townships which are not representative of the whole of Lusaka, Zambia. Perhaps another study focusing on different residential areas within Lusaka or rural areas may yield different results. Furthermore, the distances from place of residence to local clinic were established using Google maps. The accuracy of using Google maps to determine distances is not clear.

Self-screening kits for cervical cancer were considered acceptable to a majority of the study respondents, including women who have practiced screening. This indicates that self-screening can be an acceptable alternative for women under-screened for cervical cancer. Future studies can assess the implementation of self-screening as a way to help reduce the incidence of cervical cancer and the barriers to routine screening.

Considering the results of this study, some policy recommendations can be suggested. Firstly, there is a need to ensure that cervical cancer screening services are available at all clinics. Due to low levels of knowledge, perhaps having these services at all clinics may lead to a better outreach between healthcare practitioners and women who may attend the clinic even for other health reasons. Regarding vaccination, the overall interest in vaccination was a positive finding. Since, reproductive activities start very early in Zambia, it is therefore crucial to start vaccines early during adolescence. Cancer vaccines can either be included in the normal immunization schedule as cost-effective solution or community based vaccine drives can be initiated through the MoH. In general, improvements in screening attendance and vaccine acceptance along with behavior change will have a major impact in the prevention of cervical cancer in Zambia.

## Additional files

Additional file 1: Cervical cancer prevention methods questionnaire for females. The questionnaires used to collect data from women (DOCX 84 kb)

Additional file 2: Cervical cancer prevention methods questionnaire for males. Description of data: The questionnaires used to collect data from men (DOCX 80 kb)

## Abbreviations

ACEWCC: African Centre of Excellence for Women’s Cancer Control
CCPPZ: Cervical Cancer Prevention Program in Zambia
CI: Confidence Interval
CIDRZ: Centre for Infectious Disease Research in Zambia
CSO: Central Statistical Office
*df*: Degree’s of freedom
eC3: Electronic cervical cancer control
GPS: Global Positioning System
HIV: Human immunodeficiency virus
HPV: Human papillomavirus
M: Mean
MoH: Ministry of Health
N: Population/Sample size
OR: Odds Ratio
SD: Standard Deviation
SEM: Social Ecological Model
SPSS: Statistical Package for the Social Sciences
STI: Sexually Transmitted Infection
TTI: Theory of Triadic Influence
UTM: Universal Transverse Mercator
VIA: Visual Inspection with Acetic acid
WHO: World Health Organization
β: beta value
χ^2^: Chi-square
