# Supplementary material for: Knowledge, attitudes and practices of cervical cancer prevention among Zambian women and men
Source: BMC Public Health. 2019 May 4;19:508. doi: 10.1186/s12889-019-6874-2 (PMC6500583; doi:10.1186/s12889-019-6874-2)
Supplement: Supplementary file 1 — Cervical cancer prevention methods questionnaire for females. The questionnaires used to collect data from women (DOCX 84 kb) [file 12889_2019_6874_MOESM1_ESM.docx]

**CERVICAL CANCER PREVENTION MEHTODS QUESTIONNAIRE FOR FEMALES**

GPS location:

Start time:

Name of interviewer Date of interview Identification code

Instructions:

1. This questionnaire has three sections.

SECTION A: Demographics (to know more about you)

SECTION B: General (to know about your personality)

SECTION C: Cervical cancer (covers general information, screening, and vaccination)

1. Answer questions by marking an “X” in the box that represents the desired response or by circling the number that represents the desired response. For questions without answer options, write the response in the box.

Examples:

1. Gender: Male Female
2. Mark the response that represents your answer:

|  | Yes | No | Not sure |
| --- | --- | --- | --- |
| Do you know what cervical cancer is? | 1 | 2 | 3 |

**Read before starting the interview**

The purpose of this study is to determine cervical cancer prevention methods that can be practiced in Zambia. The questionnaire will be primarily assessing the views of Zambian women and men on cervical cancer screening and vaccination. There are no right or wrong answers. We are only interested in what you are thinking. Your responses will be anonymous and will never be linked to you personally. Your participation is entirely voluntary. In case you wish to decline to take part in the study, you may do so and nothing will be made against you. Thank you for your cooperation.

Kindly answer all questions, without skipping any. Be as honest as possible with your answers.

| **SECTION A: DEMOGRAPHICS** |
| --- |

1. What is your year of birth?

Year

1. What is the highest educational level that you have attained?

1 No formal education

2 Incomplete primary school

3 Complete primary school

4 Incomplete secondary school

5 Complete secondary school

6 Incomplete college, without diploma

7 Complete college, with diploma

8 Some university-level education, without degree

9 University-level education, with degree

1. Are you employed now or not?

1 Full time employee

2 Part time employee

3 Self employed

4 Retired/pensioned

5 Housewife/unemployed

6 Student

7 Other

1. During the past year, did your family: (mark all that apply)

Save money

Just get by

Spent some savings

Spent savings and borrowed money

1. Do you belong to a religion or religious denomination?

1 No (do not belong to a denomination/religion) → go to question A9

2 Catholic → go to question A6

3 Christian Protestant → go to question A6

4 Orthodox → go to question A6

5 Jew → go to question A6

6 Muslim → go to question A6

7 Hindu → go to question A6

8 Buddhist → go to question A6

9 Other → go to question A6

**If question A5 = Catholic, Christian, Orthodox, Jew, Muslim, Hindu, Buddhist or Other, continue from here**

1. Apart from weddings and funerals, about how often did you attend religious services during the past year?

1 More than once a week

2 Once a week

3 Once a month

4 Only on special holidays

5 Once a year

6 Less often

7 Never, practically never

1. How important is it to you to rely on your religious beliefs as a guide for day-to-day living?

1 Not important at all

2 Of little importance

3 Moderately important

4 Important

5 Very important

1. What kind(s) of religious activities do you practice?

|  | Yes | No |
| --- | --- | --- |
| A8.1 Do you pray before you have a meal? | 1 | 2 |
| A8.2 Apart from meal times, do you regularly pray in your home? | 1 | 2 |
| A8.3 Do you burn candles for religious purposes? | 1 | 2 |
| A8.4 Do you regularly wear clothing that is symbolic of your faith? | 1 | 2 |
| A8.5 Are the choices you make about the food you eat based on your faith? | 1 | 2 |
| A8.6 Does your physical appearance (e.g. hair cut, tattoos, piercings) symbolize your faith? | 1 | 2 |

**If question A5 = No, continue from here**

1. With whom do you live? (mark all that apply)

You live alone

Partner

Step children

Biological children

Children (not your biological/step children)

Other family members

Other (e.g. with friends)

1. How many children live with you in total?

Number

1. From the children in your household, how many are girls between 5 – 18 years old (school going)?

Number

1. From the children in your household, how many are boys between 5 -18 years old (school going)?

Number

1. How many of your biological children between 5 -18 years old (school going) live somewhere else, not with you?

Number

1. What are the characteristics of the school your child/children go to? (mark all that apply)

Private

Public

Religious

Non-religious

Boarding school

| **SECTION B: GENERAL** |
| --- |

**The following questions are general questions about your personality in terms of decision making and the control you feel you have over your life.**

1. On average during the past year, how many times have you met with other people at your or their homes or in a public place just for the sake of socializing?

1 Never, practically never

2 Once a month

3 Once a week

4 More than once a week

5 Once a day

6 More than once a day

Please rate the following questions about control and decision making.

| 1. Do you feel that you have the power to make important decisions that change the course of your life (for instance, changing jobs or moving to a different city)? | Totally unable to change life | Mostly unable to change life | Neither able nor unable to change life | Mostly able to change life | Totally able to change life |
| --- | --- | --- | --- | --- | --- |
|  | 1 | 2 | 3 | 4 | 5 |
| 1. How much control do you feel you have in making decisions that affect your everyday activities? | No control | Control over few decisions | Control over some decisions | Control over most decisions | Control over all decisions |
|  | 1 | 2 | 3 | 4 | 5 |
| 1. Some people feel they have completely free choice and control over their lives, while other people feel that what they do has no real effect on what happens to them. How much freedom of choice and control you feel you have over the way your life turns out? | No choice at all | Mostly unable to choose | Neither able nor unable | Mostly able to choose | A great deal of choice |
|  | 1 | 2 | 3 | 4 | 5 |
| 1. Some people complete the tasks they begin and others give up early. Do you consider yourself to be self-determined? | Disagree completely | Disagree | Neither agree nor disagree | Agree | Completely agree |
|  | 1 | 2 | 3 | 4 | 5 |

1. In general, who do you feel is mainly responsible for your healthcare?

1 You

2 Your family

3 Your work place

4 The local government

5 The national (Zambian) government

5 Your religious deity (God, Allah, higher power)

6 Other, namely:

1. In general, who do you feel is mainly responsible for your children’s healthcare?

1 You

2 Your partner/husband

3 Both you and your partner

4 Your extended family

5 The local government

6 The national (Zambian) government

7 Your religious deity (God, Allah, higher power)

8 Other, namely:

1. Regarding decisions made in the household, who **usually** makes decisions about healthcare for the family (for instance, spending money on medicines, getting vaccinations)? (mark one)

1 You

2 Your partner/husband

3 Both you and your partner

4 Your parents

5 Your in-laws

6 Other

| **SECTION C: CERVICAL CANCER** |
| --- |

1. Do you know what cervical cancer is?

1 Yes → go to question C2

2 No → read the introduction and go to question C5

3 Not sure → go to question C2

**If question C1 = Yes or Unsure, continue from here**

1. According to you, what causes cervical cancer? I will give you several options to choose from. More than one answer is possible. **(show card with options)**

Smoking

Starting to be sexually active at a young age

Having many sexual partners

Being pregnant at a young age

Using contraceptives

Having many pregnancies

Being old

Bad hygienic habits

Heredity, which means it’s in the family

Having a sexually transmitted disease

Unsafe sexual practice (without a condom)

Human papillomavirus (HPV) infection

Other, explain:

1. According to you, how can a person reduce the risk of getting cervical cancer? I will give you several options to choose from. More than one answer is possible. **(show card with options)**

Not smoking

Not becoming sexually active at a young age

Being faithful to one sexual partner

Not taking oral contraceptives

Hygienic practices e.g. vaginal douching (washing/cleaning the inside of the vagina)

Practicing safe sex (with a condom)

Being circumcised (male circumcision)

Being vaccinated against Human papillomavirus (HPV)

Attending regular cervical cancer screening

Other, explain:

1. The following questions are about possible **sources** of information about cervical cancer.

| Who supplied you with information about cervical cancer: | | | Can you tell me what type of information **[fill in source]** did supply: (tick all that apply) | | | | |
| --- | --- | --- | --- | --- | --- | --- | --- |
|  | Yes | No | Screening | Vaccination | Safe sex (condom, one partner) | No sex (abstinence) | Other |
| C4.1 Your husband/ partner | 1 | 2 | 1 | 2 | 3 | 4 | 5 |
| C4.2 Other family members/ friends | 1 | 2 | 1 | 2 | 3 | 4 | 5 |
| C4.3 Healthcare providers (doctor, nurse etc.) | 1 | 2 | 1 | 2 | 3 | 4 | 5 |
| C4.4 Schools | 1 | 2 | 1 | 2 | 3 | 4 | 5 |
| C4.5 Religious group (church, mosque) | 1 | 2 | 1 | 2 | 3 | 4 | 5 |
| C4.6 TV, radio | 1 | 2 | 1 | 2 | 3 | 4 | 5 |
| C4.7 Billboards, posters | 1 | 2 | 1 | 2 | 3 | 4 | 5 |
| C4.8 Print media (newspaper, magazines) | 1 | 2 | 1 | 2 | 3 | 4 | 5 |
| C4.9 Internet (social media, websites) | 1 | 2 | 1 | 2 | 3 | 4 | 5 |

***Information regarding cervical cancer for all participants***

**I am going to take a minute now to explain the most basic things about cervical cancer so that you will know what it is about.**

*Cervical cancer is a disease of the cervix, which is found at the bottom part of the womb (between womb and vagina). It is caused by an infection with a virus called Human Papillomavirus. The tissue of the cervix begins to grow and eventually, it will start bleeding. As it continues to develop, cancer can spread throughout the entire body and the woman will die.*

*Cancer of the cervix is a silent disease, this means you might have it for many years without even knowing. The Human Papillomavirus is transmitted sexually and it is very common. There are many types of Human Papillomaviruses, some cause cervical cancer, others cause genital warts: small, innocent yet very annoying deformations of the skin. Fortunately, not every woman who becomes infected develops cancer of the cervix or genital warts. Sometimes the woman is strong/healthy enough to halt the viruses. There are however viruses that are very strong/aggressive and very difficult to stop.*

*Through a gynaecologist examination called cervical screening, a healthcare provider might see the changes in the cervix and treat the disease before it becomes fatal. Unfortunately, many women in Zambia still die of cervical cancer because they do not go for regular screening.*

*There is a vaccine that prevents infection with Human Papillomaviruses, the ones that definitely cause cervical cancer or genital warts. The vaccines also offer some protection against other kinds of vaginal, anal and oral cancers. Vaccination ensures that you won’t develop cervical cancer nor genital warts caused by these viruses. The vaccine is recommended for young girls who are not yet sexually active because they have not been infected with the virus yet. The vaccine can also be used by boys. However, because of the many types of Human Papillomaviruses, even if you are vaccinated it is still necessary to get screened.*

**If question C1 = No, continue from hereafter reading information**

1. Does the topic of cervical cancer make you feel uncomfortable\nervous?

1 Not uncomfortable at all

2 A little uncomfortable

3 Rather uncomfortable

4 Very uncomfortable

**Interviewer: please rate your own observation**

1 Not uncomfortable at all

2 A little uncomfortable

3 Rather uncomfortable

4 Very uncomfortable

**I thank you that you are bearing with me. Your participation in the questionnaire on this sensitive issue is greatly appreciated. The information you are providing is important for the betterment of the country’s cervical cancer prevention program. Now to continue with the rest of the interview:**

1. The following questions are about possible **sources** of information about cervical cancer. Suppose you wanted to learn more about this topic than just the brief introduction I just supplied to you.

| Do you consider **[fill in source]** as a good source of information about cervical cancer: | Yes | No |
| --- | --- | --- |
| C6.1 Your husband/ partner | 1 | 2 |
| C6.2 Other family members/friends | 1 | 2 |
| C6.3 Healthcare providers (doctor, nurse, etc.) | 1 | 2 |
| C6.4 Schools | 1 | 2 |
| C6.5 Religious group (church, mosque) | 1 | 2 |
| C6.6 TV, radio | 1 | 2 |
| C6.7 Billboards, posters | 1 | 2 |
| C6.8 Print media (newspaper, magazines) | 1 | 2 |
| C6.9 Internet (social media, websites) | 1 | 2 |

Please answer the following questions about cervical cancer in your neighbourhood.

| 1. Do you think that cervical cancer is an important issue in your neighbourhood? | Yes | No | Not sure |
| --- | --- | --- | --- |
|  | 1 | 2 | 3 |
| 1. Is it easy to find a healthcare provider (i.e. nurse or doctor) to provide you with cervical cancer prevention services in your neighbourhood? | Yes | No | Not sure |
|  | 1 | 2 | 3 |
| 1. Cervical cancer prevention programs have been organized in my neighbourhood. | Yes, at least once | No, none | Not sure |
|  | 1 | 2 | 3 |

1. What is your estimate of the chance that you will ever get cervical cancer?

1 Very low (a 1 in 100000 shot)

2 Low (a 1 in 10000 shot)

3 Not low/not high (a 1 in 1000 shot)

4 High (a 1 in 100 shot)

5 Very high (a 1 in 10 shot)

1. In general, what are your views about Sexually Transmitted Diseases (STDs) and cancer? Please rate whether you agree or disagree with the following statements:

| To what extent do you agree or disagree with the following statements: | Completely disagree | Disagree | Neither agree nor disagree | Agree | Completely agree |
| --- | --- | --- | --- | --- | --- |
| C11.1 When someone has a sexually transmitted disease it is because of their behaviour and they deserve to be judged | 1 | 2 | 3 | 4 | 5 |
| C11.2 It is more shameful to have a sexually transmitted disease compared to having cancer | 1 | 2 | 3 | 4 | 5 |
| C11.3 It is normal to feel sorry for someone who has cancer | 1 | 2 | 3 | 4 | 5 |
| C11.4 It is shameful to have cancer | 1 | 2 | 3 | 4 | 5 |
| C11.5 When someone has cancer it means they will die | 1 | 2 | 3 | 4 | 5 |

***Cervical cancer screening***

1. Do you know where to go to get cervical screening?

1 Yes

2 No

3 Not sure

1. Do you know about screening services in government clinics?

1 Yes

2 No

1. Do you know someone who has gone for cervical cancer screening?

1 Yes

2 No

1. Out of every 10 women in your neighbourhood, how many do you think attend cervical screening?

1 None of them

2 Less than 5 but not none

3 Half of them

4 More than 5 but not all

5 All of them

1. Have you ever had cervical screening?

1 Yes → go to question C17

2 No →go to question C18

3 Not sure → go to question C18

1. Did someone ever offer to accompany you to cervical screening?

1 Yes → go to question C19

2 No → go to question C19

**If question C16 = No or Not sure, continue from here**

1. If you never went for screening or you are not sure. Can you tell me what keeps you from going?

| To what extent do you agree or disagree with the following statements: | Completely disagree | Disagree | Neither agree nor disagree | Agree | Completely agree |
| --- | --- | --- | --- | --- | --- |
| C18.1 Finding time to go for screening is difficult | 1 | 2 | 3 | 4 | 5 |
| C18.2 Transportation to go for screening is difficult to find | 1 | 2 | 3 | 4 | 5 |
| C18.3 It’s too expensive to get to the clinic | 1 | 2 | 3 | 4 | 5 |
| C18.4 I am nervous about being screened | 1 | 2 | 3 | 4 | 5 |
| C18.5 I expect cervical screening to be painful | 1 | 2 | 3 | 4 | 5 |

**If question C17 = Yes or No, continue from here**

1. Which of the following **sources** would approve or disapprove with you on going for cervical cancer screening? Does their opinion affect your final decision?

|  | Will **[fill in source]** approve/disapprove: | | | | | Does it affect your decision: | |
| --- | --- | --- | --- | --- | --- | --- | --- |
|  | Completely disapprove | Disapprove | Neither approve nor disapprove | Approve | Completely approve | Yes | No |
| C19.1 Husband/partner | 1 | 2 | 3 | 4 | 5 | 1 | 2 |
| C19.2 Other family members | 1 | 2 | 3 | 4 | 5 | 1 | 2 |
| C19.3 Friends | 1 | 2 | 3 | 4 | 5 | 1 | 2 |
| C19.4 Religious group (church) | 1 | 2 | 3 | 4 | 5 | 1 | 2 |

1. If you had the chance to screen yourself at home with a self-screening kit, would you be willing to do it this way if it was easy to do?

1 Yes

2 No

3 Not sure

1. If you had to pay, how much would you pay to buy a self-testing kit for routine cervical cancer screening?

1 I am not able to pay

2At most10 Kwacha

3 At most K20

4 At most K30

5 At most K40

6 At most K50

7 Above K50

8 Not sure

1. At what age do you think women should start screening?

Number

1. What age do you think women stop screening?

Number

1. What do you think of the following statement: When the results of cervical cancer screening show that something is wrong, the chance for cure is high

1 Completely disagree

2 Disagree

3 Neither agree nor disagree

4 Agree

5 Completely agree

***Cervical cancer vaccination***

1. Did you know about free cervical cancer vaccination for school age girls?

1 Yes

2 No

1. Schools in my neighbourhood have participated in the cervical cancer vaccination program

1 Yes, at least one

2 No, none

3 Not sure

1. Do you know someone who has gone for cervical cancer vaccination?

1 Yes

2 No

1. Did you have one or more of your daughters vaccinated against cervical cancer?

1 Yes, I have vaccinated my daughter(s) → go to question C30

2 No → go to question C29

**If question C28 = No, continue from here**

1. If not, would you have your daughter(s) vaccinated in the future?

1 No

2 Yes, if given a chance

3 I do not have a daughter, but if I did I would vaccinate her

4 I do not have a daughter, but if I did I would not vaccinate her

**If question C28 = Yes, continue from here**

1. Some countries have allowed the vaccination of boys as well as girls seeing that the vaccine not only prevents cervical cancer but it can also prevent the development of oral cancers, genital warts, etc. Knowing this information, would you vaccinate your son(s) against the carrier of cervical cancer, if that option was available to you?

1 No

2 Yes, if given a chance

3 I do not have a son, but if I did I would vaccinate him

4 I do not have a son, but if I did I would not vaccinate him

1. Which of the following **sources** would approve or disapprove with you on cervical cancer vaccination? Does their opinion affect your final decision?

|  | Will **[fill in source]** approve/disapprove: | | | | | Does it affect your decision: | |
| --- | --- | --- | --- | --- | --- | --- | --- |
|  | Completely disapprove | Disapprove | Neither approve nor disapprove | Approve | Completely approve | Yes | No |
| C31.1 Your child | 1 | 2 | 3 | 4 | 5 | 1 | 2 |
| C31.2 Husband/partner | 1 | 2 | 3 | 4 | 5 | 1 | 2 |
| C31.3 Other family members | 1 | 2 | 3 | 4 | 5 | 1 | 2 |
| C31.4 Friends | 1 | 2 | 3 | 4 | 5 | 1 | 2 |
| C31.5 Religious group (church) | 1 | 2 | 3 | 4 | 5 | 1 | 2 |

The vaccine is recommended to be taken before one becomes sexually active. However, it can be administered from the age of 9 to 26 years old. Considering this age range, answer the following questions:

1. At what age would you consider to have your daughter(s) vaccinated? Briefly explain your reason why.
2. At what age would you consider to have your son(s) vaccinated? Briefly explain your reason why.

C34. What are the reasons for vaccinating or not vaccinating your children? Please rate how much you agree or disagree with the following statements:

End time:

|  | Completely disagree | Disagree | Neither agree nor disagree | Agree | Completely agree |
| --- | --- | --- | --- | --- | --- |
| C34.1 Using vaccines shows a lack of faith in the healing power of God | 1 | 2 | 3 | 4 | 5 |
| C34.2 The knowledge to create vaccines was given to people by God or a higher power | 1 | 2 | 3 | 4 | 5 |
| C34.3 The cervical cancer vaccine is harmful to health | 1 | 2 | 3 | 4 | 5 |
| C34.4 If you vaccinate young girls they will be sexually active too early | 1 | 2 | 3 | 4 | 5 |
| C34.5 Vaccination is an effective way of preventing cervical cancer | 1 | 2 | 3 | 4 | 5 |
| C34.6 It is morally wrong to vaccinate girls for cervical cancer | 1 | 2 | 3 | 4 | 5 |
| C34.7 It is morally wrong to vaccinate boys for cervical cancer | 1 | 2 | 3 | 4 | 5 |

C35. Would you like to take part in a small group discussion about your views on cervical cancer? If yes, please provide your phone number and state when you have free time (day(s) and time range)

**We have been discussion a tough subject. Here is a leaflet with further information on cervical cancer prevention from the local clinic. Thank you once again for your participation. Have a nice day.**

**OBSERVATION BY INTERVIEWER**

1. How interested was the respondent during the interview?

1 Respondent was very interested

2 Respondent was somewhat interested

3 Respondent was not interested

1. Did the interview take place in privacy or not?

1 There were no other people around who could follow the interview

2 There were other people around who could follow the interview

3 There were other people around who intervened/answered questions instead of the respondent

1. Did the interview run smoothly or not?

1 Yes→ go to question D5

2 No→ go to question D4

1. If the interview did **not** run smoothly, what problems were met?
2. Any other remarks about the interview:
